# Societies at risk: the association between conflict intensity and population health indicators in Venezuela

**DOI:** 10.1186/s12963-025-00377-x

**Published:** 2025-04-10

**Authors:** Emilia Olson, MHD Bahaa Aldin Alhaffar, Anneli Eriksson

**Affiliations:** 1 Department of Global Public Health, Karolinska Institute, Stockholm, Sweden; 2 School of Medicine, University of South Carolina, Greenville, SC USA; 3 Diseases Program, European Center for Diseases Control and Prevention, Stockholm, Sweden

**Keywords:** Armed conflict, Venezuela, Panel data study, Malaria, Heart disease, Infant mortality

## Abstract

**Background:**

Venezuela present a complex political and humanitarian context as the country is suffering from internal conflict and socio-political crisis which led to the deterioration of the health services, hyperinflation, and migration crisis, and this presents a unique case to explore the impact of conflict intensity on health outcomes. This study investigates potential relationships between conflict intensity and key health indicators in Venezuela from 2001 to 2016, focusing on malaria, heart disease mortality, and infant mortality rates.

**Methods:**

Employing an ecological panel data analysis approach, this research analyzes state-year level data from the Uppsala Conflict Data Program and the Venezuelan Health Observatory. The study focuses on assessing if and how conflict intensity influences malaria incidence, heart disease mortality rates, and under-1 infant mortality rate across Venezuelan regions, using panel data regression with fixed effects for state and year.

**Results:**

The study identifies a statistically significant correlation between conflict intensity high estimate and higher rates of infant mortality and heart disease mortality. Interestingly, no significant correlation was found between conflict intensity and malaria incidence. These findings suggest the multifaceted impacts of armed conflicts on health outcomes, indicating that while some health indicators deteriorate with rising conflict intensity, others may not exhibit direct correlations.

**Conclusion:**

This study underscores the complex relationship between armed conflict intensity and health outcomes in Venezuela, highlighting significant correlations with infant mortality and heart disease mortality, but not with malaria incidence or the conflict death best estimate. The best estimate from UCDP didn’t show correlation, while the high estimate showed significant correlation. The limitations posed by the UCDP database constraints, and the absence of recent health data publication invite further research to explore the nuanced impacts of conflict on health.

**Supplementary Information:**

The online version contains supplementary material available at 10.1186/s12963-025-00377-x.

## Introduction

In 2023, the world has witnessed an unprecedented surge in armed conflicts, surpassing any year since the Second World War, with a quarter of the world’s population living in conflict-affected areas [1]. This escalation of armed confrontations has significantly contributed to the global burden of disease, reversing the previous trend of declining conflict-related mortality [1]. The Uppsala Conflict Data Program (UCDP) reported a stark figure of 311,225 conflict deaths in 2022, underscoring the critical need for focused academic exploration into the multifaceted impacts of armed conflicts on health [2]. These conflicts, beyond the immediate toll of direct deaths, indirectly affect morbidity and mortality, influencing foundational aspects of society such as water availability, healthcare systems, and employment [3, 4].

Previous studies have established direct and indirect link between armed conflict and worsening health outcomes, a panel data analysis including data from 42 countries found increased conflict intensity correlated with higher mortality, but significance depended on adjustments for healthcare functionality [5]. Similarly, A study on the Syrian crisis -which is a protracted conflict- found a significant increase in maternal and child mortality due to the prolonged conflict, primarily driven by healthcare system collapse and worsening socioeconomic conditions [6]. In South Sudan, a recent analysis showed that higher conflict intensity correlated with child malnutrition and under-five mortality, indicating the effect of conflict on health outcome in correlation with other confounding variables [7].

Venezuela, officially the Bolivarian Republic of Venezuela, is a country on the northern coast of south America, with a population of 29 million (according to 2022 estimation) [8], and crude mortality rate of 8.1%, and expected yearly death of 240 thousand [9]. The crisis in Venezuela represents a complex interaction between armed conflict and health status. The country is ranked among the lowest in Latin America for health, democracy, and human rights, Venezuela has faced a spiraling crisis since the election of Hugo Chavez in 1998, further exacerbated under President Maduro’s tenure since 2014 [10, 11]. This period has been marked by hyperinflation, economic depression, and a complex humanitarian emergency, leading to significant emigration and an alarming rate of intentional homicides. ​Before the onset of the political and economic crisis in the early 2000s, Venezuela’s healthcare system was improving [12]. In 2000, the health expenditure was 5.2% of GDP, and the physician density was approximately 1.9 per 1,000 people [13]. The infant mortality rate had declined from 23 per 1,000 live births in 1995 to 21 per 1,000 in 2000, and malaria incidence remained relatively controlled, and non-communicable diseases such as heart disease followed patterns like other Latin American countries indicating some improvement in the health status of the general population [13]. However, the healthcare sector faced systemic challenges, which worsened significantly as the crisis deepened after 2001 [14]. The Venezuelan context, characterized by one-sided violence predominantly perpetrated by the government against its citizens, presenting the interaction between conflict intensity and health indicators [15].

Despite the acknowledged global impact of armed conflicts on population health, the specific consequences within the Venezuelan context remain under-explored. The direct repercussions, such as injuries and trauma from warfare, are only a fraction of the broader health crisis [16]. Indirect effects, including the degradation of healthcare infrastructure and the social determinants of health, like forced migration and internal displacement, further complicate the health landscape in conflict-affected regions [4, 17]. In Venezuela, a nation already grappling with many health challenges, the ongoing conflict exacerbates existing vulnerabilities, making it imperative to assess how key health indicators are affected [18]. Studies that are examining war-related injuries and mental health issues, provide valuable insights but fail to encompass the full spectrum of health consequences resulting from the conflict [19].

This study explores the health impacts of armed conflict in Venezuela from 2001 to 2016, it assesses the effects of conflict intensity on malaria, heart disease mortality, and infant mortality across Venezuelan regions. This methodological approach offers insights into the conflict-health relationship, informing policy and healthcare strategies in conflict-affected areas. This will help in understanding the conflict’s health implications in Venezuela but could also provide a grounded basis for policy formulation and healthcare interventions in similar conflict-affected settings.

### Aim of the research

The aim of the research is to investigate the potential relationship between conflict intensity in Venezuela from 2001 to 2016 and on key health outcomes, specifically focusing on malaria incidence, heart disease mortality, and infant mortality rates on national and subnational level.

## Methods and materials

### Study design

The study utilized an ecological panel data analysis approach to explore the effects of conflict intensity on three distinct health outcomes in Venezuela from 2001 to 2016. By focusing on a state-year level analysis, the research aims to elucidate the impacts of varying conflict intensities on malaria incidence, heart disease mortality rates, and under-1 infant mortality across the nation’s states. These particular health indicators were selected to represent significant aspects of public health: infectious diseases, through malaria cases; chronic illnesses, via heart disease mortality; and child health outcomes, as indicated by infant mortality rates. This selection enables a comprehensive evaluation of the country’s health challenges in the face of conflict. Different frameworks describe the direct and indirect health consequences of conflict, Murray et al. (2002) explore the increase diseases burden during time of conflict [4], The WHO Social Determinants of Health Framework (2008) further explains how conflict acts as a structural determinant, influencing access to healthcare, nutrition, and living conditions, which directly impact health outcomes [20]. Additionally, Garry & Checchi (2020) highlight the cascading effects of armed conflict on population health, reinforcing that infectious disease risk, chronic health outcomes, and maternal-child mortality are among the most sensitive indicators of conflict-driven health deterioration [3]. These frameworks provide a strong theoretical foundation for selecting these three health indicators to assess the impact of conflict intensity on population health in Venezuela.

Data is broken down by state and year to provide a detailed geographic and temporal understanding of these health outcomes. The sample was chosen based on the compatibility of the two data sets: from UCDP and the OVS. In 2016, the OVS halted publications with population health data. This factor determined the final year of the study.

### Data collection and source

For this study, data collection was meticulously carried out from two primary sources:

- *Uppsala conflict data program (UCDP)* This database Provided detailed armed conflict intensity data, which is measured by the number of direct conflict deaths by state and year [2]. This data is collected by the UCDP and uses both conflict death best estimate (CDBE), and conflict death high estimate (CDHE). Given the discrepancy between the conflict deaths reported by UCDP and those reported by other agencies—with UCDP figures generally being lower—the high estimate is considered potentially more reflective of the real situation [21]. Data regarding all types of conflict (state-based, one-sided, non-state) were included in the study. Conflict intensity can be defined using many direct and indirect variables, including economic, social, and public health impacts [22, 23]. However, this study adopts the UCDP’s approach which present the conflict intensity as the number of conflict related death. The conflict intensity is presented using the case count of the conflict related death, and by using the death rate which is calculated as the number of conflict-related deaths per 100,000 inhabitants, based on recent population data [24].
- *Venezuelan health observatory* Offered comprehensive data on health outcomes, specifically focusing on malaria incidence, heart disease mortality, and infant mortality rates across Venezuela’s regions throughout the duration of the study.
  - *Malaria cases* This indicator helps in assessing the spread and control of infectious diseases, with malaria serving as a key concern in conflict-affected regions due to disruptions in public health services and increased exposure to mosquito breeding sites, related to displacement. Measured by the number of new malaria cases reported per year, indicating the incidence of the disease within the population, per state. In this study, Malaria was presented using the incidence, and the malaria rate.
  - *Heart disease deaths* By analyzing mortality rates attributed to heart disease, the study investigates the broader effects of conflict on chronic health conditions. Heart diseases often require long-term care and management, which can be severely impacted by the instability and resource constraints posed by conflict situations. Heart diseases encompass a range of conditions affecting the heart’s functionality, including rheumatic heart diseases, hypertensive heart disease, heart disease related to chronic kidney conditions, and other forms of heart diseases as categorized under the International Classification of Diseases (ICD) codes I05-I09, I11, I13, and I21-I51. Heart diseases in this study was presented using the Deaths, which is the total number of heart diseases deaths per state/year, and the mortality rate which is the annual number of deaths attributed to heart-related conditions per 100,000 individuals, serving as an indicator of cardiovascular health challenges.
  - *Under-1 infant deaths* The inclusion of infant mortality as an indicator provides insight into the health system’s capacity to ensure the well-being of the most vulnerable population—infants. High infant mortality rates can indicate significant deficiencies in maternal and child health services, nutrition, and overall public health infrastructure. Under-1 infant deaths as presented in this study using the mortality which is the total number of under-1 deaths per state/year, and using infant mortality rate which is the annual number of deaths of infants under one year of age per 1,000 live births.

### Statistical analysis

The statistical analysis involved descriptive statistics to outline trends and magnitudes of armed conflict and health outcomes. Panel data regression with fixed state-year effects was applied to assess the influence of conflict intensity on health variables, utilizing STATA V.12 for data analysis. The fixed-effects model was chosen to account for **time-invariant characteristics** within each state, ensuring that the estimated effects of conflict intensity are not biased by regional differences in healthcare infrastructure, economic conditions, or demographic factors. This approach allowed for controlling unobserved heterogeneity across states and over time, providing a nuanced understanding of the relationships under study. Each model produced coefficient estimates indicating the relationship between conflict intensity and health outcomes where p-values and significance levels was used to assess statistical relevance.

The primary model specification is:

$$Y_{it} = \beta_{0} + \beta_{1} {\text{Conflict}}\;{\text{Intensity}}_{it} + \beta_{2} X_{it} + \gamma_{t} + \delta_{i} + \in_{it}$$

where:

- *Y*_*it*_ = Health outcome (malaria incidence, heart disease mortality, or infant mortality) for state i at time t.
- *Conflict Intensity *_*it*_ = Conflict intensity measure (Conflict Deaths Best Estimate (CDBE) or Conflict Deaths High Estimate (CDHE)).
- *X*_it_ = Control variables (e.g., state-level population).
- γ_t_ = Time fixed effects (captures national-level trends over time).
- δ_i_ = State fixed effects (controls for unobserved, time-invariant characteristics of each state).
- ε_it_ = Error term.

## Results

### Descriptive analysis of the research variables

The analysis of conflict-related deaths from 2001 to 2016 reveals a variable pattern of conflict intensity. The best estimate’s total was 434 deaths, whereas the high estimate’s sum was 810 deaths. The rate per 100.000 fluctuated annually, with the best estimate mean ranging from 0.06 to 0.26 per 100,000, and the high estimate from 0.09 to 0.41 per 100,000. The aggregate rates per 100,000 population over the entire period were 1.88 for the best estimate and 3.51 for the high estimate. Table 1, Appendix 1.

**Table 1:** Descriptive analysis of the research variables (2001–2016)

	Sum	Mean	Min	Max	SD
Conflict deaths best estimate	434	1.13	0	27	2.56
Conflict deaths high estimate	810	2.11	0	39	3.91
Malaria cases	1,002,799	2,611.46	0	177,619	12,844.82
Malaria incidence rate	–	546.46	0	15,213.44	1,944.27
Heart diseases deaths	377,877	1,124.63	34	5,506.00	970.54
Heart disease mortality rate	–	105.90	38.23	168.47	23.30
Infant deaths (under 1 year)	145,371	378.57	47	1818	306.96
Infant mortality rate	–	41.43	7.40	150.43	19.10

Total number of Malaria cases in the same period was 1,002,799 cases with standard deviation of [12,844.82), and the malaria incidence rate per 100,000 population was on average 546.46. The total number of heart diseases death was 377,877 (SD = 970.54), and the heart diseases mortality rate was 105.90. Finally, the under 1 infant death sum was 145,371 (SD = 306.96), and the infant mortality rate was on average 41.43 (SD = 19.10). Table 1

**Fig. 1:**
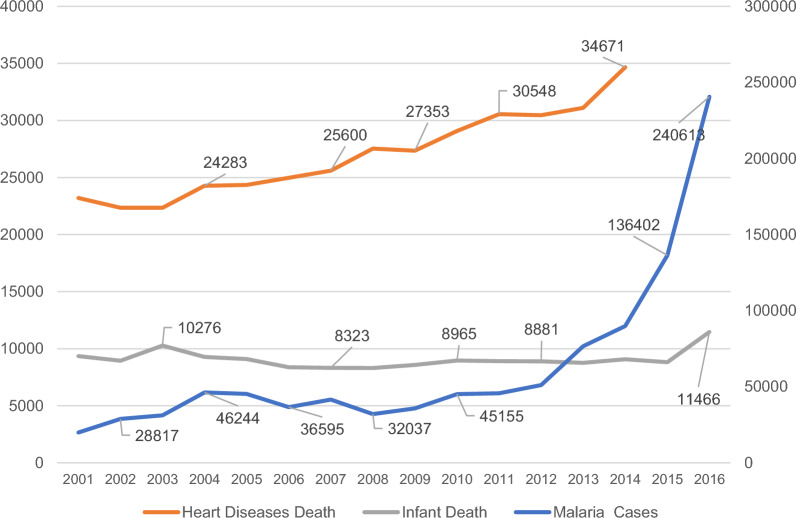
Annual number of malaria cases, heart diseases deaths, and infant deaths

Figure 1 represents the total number of cases per year for malaria, heart disease mortality and infant mortality. Both malaria and heart disease mortality indicate a progressive increase in both conditions. Malaria cases escalated after 2013, reaching an apex in 2016 with 240,613 reported instances. Heart disease mortality followed a more gradual upward trend, with year-on-year increases culminating in the highest figure of 34,671 cases in 2016. These visual trends denote a concerning rise in these health conditions towards the end of the studied timeframe. Infant mortality displayed a variable pattern but spiked to 11,466 cases in 2016.

**Fig. 2:**
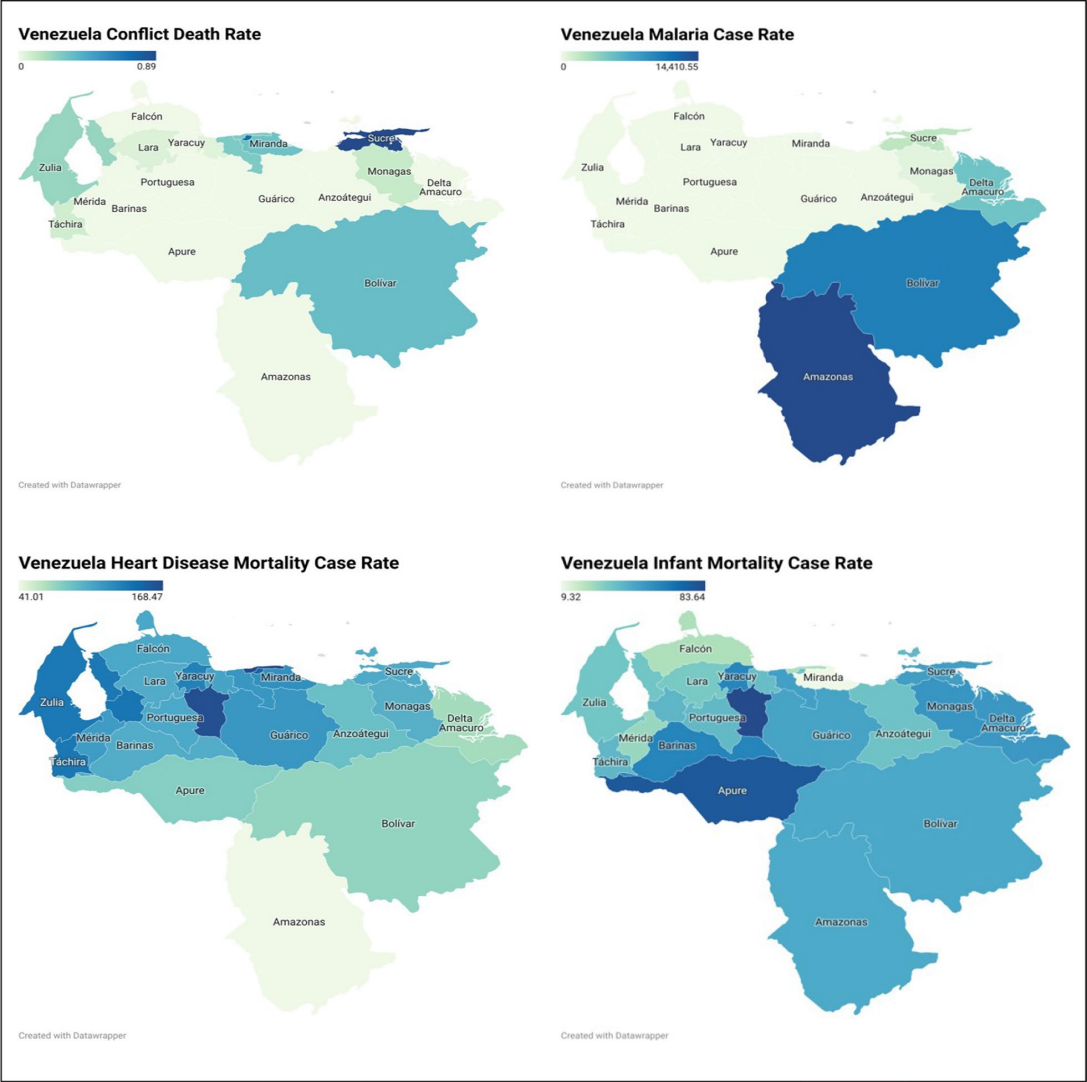
geographical representation of the conflict intensity, Malaria, Heart disease mortality, and infant mortality

Geographic representation of the data showed that many conflict related deaths are concentrated in certain states such as Sucre, the Capital, and Bolivar. Additionally, certain states have no recorded conflict deaths including Amazonas and Delta Amacuro. Malaria cases were the highest in the Amazonas, Bolivar, and Delta Amacuro. Heart disease mortality was higher in the north of Venezuela, while infant mortality spread across the county with Apure being the highest. Figure 2

**Fig. 3:**
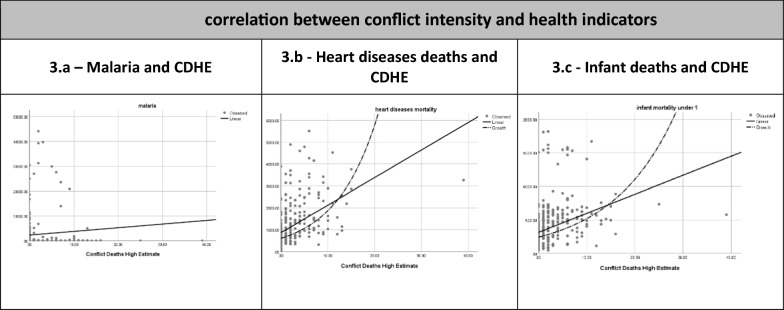
correlation between conflict intensity and health indicators

Figure 3 illustrates the correlations between conflict intensity and health indicators. For malaria, the relationship was not significant with high variability in mean cases across the conflict death high estimate. Heart diseases mortality shows a significant positive correlation with conflict deaths, indicating higher disease counts in relation to increased conflict-related deaths. Infant mortality exhibits a similarly variable but generally positive trend, suggesting that as the conflict death high estimate rises, mean infant mortality does as well. These visual patterns align with the statistical correlations previously noted, confirming significant associations for heart diseases mortality and infant mortality with conflict intensity.

A linear regression model was employed to examine the link between conflict intensity and health-related variables. This analysis revealed a statistically significant correlation between infant mortality rates and Conflict Deaths High Estimate (CDHE), with a 95% confidence interval, highlighting a significant association at a p-value of 0.042. Furthermore, a notable correlation was identified between infant mortality and Conflict Deaths at Best Estimate (CDBE), alongside a connection between heart disease mortality rates and CDHE at a lesser confidence level of 90%, indicated by p-values of 0.065 and 0.060, respectively. Table 2

**Table 2:** Panel data results of armed conflict and health indicators with fixed effects for region and year and controlling for population

Health variables	Malaria cases		Heart disease deaths		Infant morality	
Conflict deaths	Best	High	Best	High	Best	High
Coefficient	879.8	568.9	− 12.53	10.14*	20.09*	15.22**
Robust standard error	(514.9)	(371.3)	(7.650)	(5.145)	(10.37)	(7.075)
P-value	0.100	0.139	0.115	0.060*	0.065*	0.042**
Observations	400	400	350	350	400	400
R-squared	0.462	0.460	0.773	0.774	0.297	0.326

^***^ *p* < 0.01, ** *p* < 0.05, * *p* < 0.1

Conversely, no significant correlation emerged between malaria incidence and conflict fatalities, whether CDBE or CDHE, nor between heart disease mortality and CDBE. The coefficients for the variables were 20.09 for infant mortality and CDBE, 15.22 for infant mortality and CDHE, and -12.53 and 10.14 for heart disease mortality with CDBE and CDHE, respectively, showcasing the statistical power of the associations. Table 2

The regression analysis reveals a statistically significant association between conflict intensity and infant mortality, where a one-unit increase in Conflict Deaths High Estimate (CDHE) correlates with a 15.22 increase in infant deaths per 100,000 live births (*p* = 0.042). Additionally, heart disease mortality shows a marginally significant relationship with CDHE, with an increase of 10.14 deaths per 100,000 (*p* = 0.060), indicating potential disruptions in chronic disease management and emergency care in conflict-affected regions. Despite the positive regression coefficient, no significant association was observed between conflict intensity and malaria incidence (*p* > 0.1), suggesting that factors beyond direct conflict exposure, such as vector control programs and environmental conditions, may have a stronger influence on malaria transmission. Table 2

## Discussion

The enduring conflict in Venezuela has precipitated a complex humanitarian crisis, profoundly impacting various aspects of society, including public health [25]. This crisis has disrupted the fabric of everyday life for millions, leading to the degradation of healthcare infrastructure, shortages of essential medicines and medical supplies, and a consequent rise in morbidity and mortality. [11, 19]. Our study embarked on an examination of the relationship between conflict intensity and pivotal population health indicators within the Venezuelan context. Utilizing an ecological panel regression approach, the research sought to investigate whether an association exists between the conflict intensity and key health outcomes, namely malaria incidence and rate, heart disease deaths and mortality rate, and infant (under 1) deaths and mortality rate. The endeavor was underpinned by a hypothesis that heightened conflict intensity might correlate with adverse health outcomes, reflecting the broader ramifications of conflict on societal well-being and health infrastructure [3, 4].

In the analysis, a significant positive correlation was observed between conflict intensity (high estimate) and both heart disease deaths and infant deaths, underscoring the detrimental impact of conflict on these health outcomes [26, 27]. This is consistent with existing literature that suggests heightened conflict scenarios often disrupt healthcare services and exacerbate risk factors associated with non-communicable diseases and adverse maternal and child health conditions [6]. Page et al. in 2019 has documented the deterioration of Venezuela’s healthcare infrastructure amidst the crisis leading to the increase of chronic health conditions as the access to healthcare became more difficult [25]. In contrast, the relationship between malaria incidence and conflict intensity did not align with the expected positive correlation. This nuance in the findings could be attributed to the complexities of malaria diagnosis and reporting in conflict-affected settings. While many studies has delineated the exacerbation of malaria risks in such contexts [28–30], noting both barriers to and strategies for malaria control [31, 32], there exists a spectrum of outcomes. Certain reports have unveiled a negative correlation between conflict levels and malaria risk [33], this could be attributed to the negative effect of conflict on the diagnosis of malaria cases, which in terms lead to a negative correlation with the conflict intensity [31, 34], and those results correspond with the outcome of our analysis. While other studies find a strong positive association between the conflict intensity and malaria cases, as conflict settings affect the treatment of malaria, and increase the transmission rate [29, 35].

The observed correlations between conflict intensity and specific health outcomes underscore the profound consequences that armed conflicts have on population health [4, 6]. This emphasizes the critical need for international health policies and interventions to prioritize healthcare access, emergency medical care, and the reinforcement of health systems in areas besieged by conflict [3, 35]. Such strategies are paramount not only in mitigating the immediate health impacts of conflicts but also in safeguarding against the long-term deterioration of public health infrastructure [36].

The weak or non-significant association observed in our study between conflict intensity and specific health outcomes may be partly attributed to the limited sample size of conflict-related deaths derived from the UCDP database. Additionally, it is possible that other variables mediated by the conflict, such as economic crises or migration issues, have a more significant impact on health outcomes. While providing verified conflict data, UCDP database is likely underestimates the true scale of conflict-related fatalities in Venezuela by excluding different cases due to its stringent verification criteria. Furthermore, the Venezuelan government’s discontinuation of health-data publications after 2016 severely restricts our analysis to a period before the crisis reportedly intensified [2].

This study can give an example of the effect of conflict intensity in Venezuela on the non-communicable diseases (NCDs) and child health, reflecting the adverse effect of the long-term instability on the population health outcomes. However, the gap in the data challenges the ability to comprehensively assess the ongoing impact of conflict on health and underscores the potential to underestimate the actual effects. This motivates international cooperation to address the healthcare needs arising from conflict, underscoring the critical importance of reinstating comprehensive health monitoring and support systems.

The findings of this study provide important insights for public health policies in conflict-affected settings. Conflict can cause direct and indirect health consequences which will be translated into excess mortality. Those correlation between the different variables shows the interlinked, multi-layered nature of conflict settings and how they contribute significantly to the overall burden of diseases in any conflict-affected country [5]. These findings can inform decision-makers by guiding resource allocation, prioritizing maternal and chronic disease healthcare in humanitarian response plans, and reinforcing the importance of resilient health systems that can withstand conflict-induced disruptions. Future policies should emphasize maintaining essential health services, integrating conflict-sensitive health planning, and securing long-term investments in health infrastructure to mitigate the consequences of conflict on population health.

This study has several limitations, the measurement of conflict intensity relies on UCDP data, which may underestimate conflict deaths due to strict verification criteria and underreporting, limiting the statistical power of our analysis. Additionally, unobserved confounders such as economic decline, migration, healthcare collapse, and food insecurity could not be fully accounted for due to data limitations, restricting causal interpretation despite the use of state and year fixed effects. The health data, sourced from the Venezuelan Health Observatory, ceased publication after 2016, preventing analysis of the crisis’s long-term impact. Moreover, reporting biases and data inconsistencies may have affected accuracy, particularly for malaria incidence, where diagnostic and reporting capacity likely deteriorated. Future research should integrate alternative conflict datasets, additional control variables, and extended time frames to improve robustness.

## Conclusion

The result of the research found a significant correlation between increased conflict intensity and rising infant deaths and heart diseases deaths in Venezuela for the period 2001–2016, signaling an alarming degradation in health conditions following the ongoing crisis. This crisis led to low number of conflict related deaths, which was represented in the small sample size of the research database. This highlights the need for more comprehensive research, incorporating wider data sources to fully ascertain the impacts of conflict on health outcomes. Future studies should consider the roles of economic instability, healthcare accessibility, and migration patterns to enrich insights.

## Supplementary Information

Supplementry meterial 1.

Supplementry meterial 2.

## Data Availability

No datasets were generated or analysed during the current study.
