# Supplementary material for: Societies at risk: the association between conflict intensity and population health indicators in Venezuela
Source: Popul Health Metr. 2025 Apr 10;23:14. doi: 10.1186/s12963-025-00377-x (PMC11983831; doi:10.1186/s12963-025-00377-x)
Supplement: Supplementary file 1 — Supplementry meterial 1. [file 12963_2025_377_MOESM1_ESM.docx]

**Appendix:**

| **Appendix 1 – Descriptive analysis of the research variables per year** | | | | | | | | | | |
| --- | --- | --- | --- | --- | --- | --- | --- | --- | --- | --- |
|  | **CDBE^1^** | **CDBE/Rate** | **CDHE^2^** | **CDHE/Rate** | **MAL^3^** | **MAL/Rate^4^** | **HDD^5^** | **HDM/Rate^6^** | **IFU1D^7^** | **IFU1/Rate^8^** |
|  | **Sum** | **Mean** | **Sum** | **Mean** | **Sum** | **Mean** | **Sum** | **Mean** | **Sum** | **Mean** |
| **2001** | 27 | 0.09 | 60 | 0.21 | 19904 | 314.43 | 23210 | 95.68 | 9353 | 47.83 |
| **2002** | 27 | 0.11 | 39 | 0.15 | 28817 | 358.72 | 22349 | 95.00 | 8949 | 44.21 |
| **2003** | 25 | 0.09 | 53 | 0.17 | 31195 | 692.50 | 22349 | 95.00 | 10276 | 51.05 |
| **2004** | 31 | 0.14 | 67 | 0.24 | 46244 | 843.33 | 24283 | 102.62 | 9276 | 47.42 |
| **2005** | 30 | 0.10 | 45 | 0.16 | 45328 | 604.85 | 24353 | 102.32 | 9093 | 46.60 |
| **2006** | 22 | 0.13 | 28 | 0.15 | 36595 | 475.04 | 24977 | 104.28 | 8371 | 41.41 |
| **2007** | 14 | 0.05 | 33 | 0.13 | 41570 | 588.99 | 25600 | 106.95 | 8323 | 40.54 |
| **2008** | 27 | 0.10 | 63 | 0.20 | 32037 | 453.20 | 27542 | 115.19 | 8307 | 40.95 |
| **2009** | 25 | 0.12 | 63 | 0.24 | 35703 | 329.70 | 27353 | 113.67 | 8577 | 42.15 |
| **2010** | 22 | 0.08 | 51 | 0.17 | 45155 | 373.78 | 29078 | 120.40 | 8965 | 43.13 |
| **2011** | 10 | 0.03 | 21 | 0.08 | 45743 | 260.18 | 30548 | 104.70 | 8900 | 34.14 |
| **2012** | 33 | 0.05 | 64 | 0.13 | 51050 | 292.82 | 30465 | 105.35 | 8881 | 36.55 |
| **2013** | 6 | 0.05 | 42 | 0.12 | 76621 | 464.24 | 31099 | 110.25 | 8757 | 36.34 |
| **2014** | 44 | 0.09 | 50 | 0.10 | 89822 | 508.97 | 34671 | 111.25 | 9065 | 33.15 |
| **2015** | 32 | 0.07 | 37 | 0.08 | 136402 | 826.08 |  |  | 8812 | 33.63 |
| **2016** | 59 | 0.14 | 94 | 0.21 | 240613 | 1356.55 |  |  | 11466 | 43.83 |
| **1- CDBE: Conflict death best estimate, 2- CDHE: conflict death high estimate, 3- MAL: malaria incidence, 4-malaria rate, 5-HDD: heart diseases deaths, 6- heart disease mortality rate 7- IFU1D: infant under 1 deaths, 8- IFU1M: infant under 1 mortality rate** | | | | | | | | | | |
