# Supplementary material for: Societies at risk: the association between conflict intensity and population health indicators in Venezuela
Source: Popul Health Metr. 2025 Apr 10;23:14. doi: 10.1186/s12963-025-00377-x (PMC11983831; doi:10.1186/s12963-025-00377-x)
Supplement: Supplementary file 2 — Supplementry meterial 2. [file 12963_2025_377_MOESM2_ESM.docx]

| **Appendix 2 – Descriptive analysis of the research variables per state** | | | | | | | | | | |
| --- | --- | --- | --- | --- | --- | --- | --- | --- | --- | --- |
|  | **CDBE^1^** | **CDBE/Rate** | **CDHE^2^** | **CDHE/Rate** | **MAL^3^** | **MAL/Rate^4^** | **HDD^5^** | **HDM/Rate^6^** | **IFU1D^7^** | **IFU1/Rate^8^** |
|  | **Sum** | **Mean** | **Sum** | **Mean** | **Sum** | **Mean** | **Sum** | **Mean** | **Sum** | **Mean** |
| **Amazonas** | 0 | 0.00 | 0 | 0.00 | 131037 | 7978.05 | 733 | 58.57 | 1289 | 86.81 |
| **Anzoátegui** | 50 | 0.25 | 83 | 0.41 | 3325 | 16.48 | 14595 | 79.35 | 5825 | 26.70 |
| **Apure** | 8 | 0.13 | 9 | 0.15 | 1605 | 23.13 | 4890 | 86.07 | 3706 | 54.31 |
| **Aragua** | 33 | 0.13 | 69 | 0.27 | 4 | 0.02 | 22919 | 108.05 | 8120 | 32.64 |
| **Barinas** | 1 | 0.01 | 2 | 0.01 | 1313 | 12.86 | 9452 | 99.33 | 4920 | 43.53 |
| **Bolívar** | 36 | 0.17 | 64 | 0.31 | 744164 | 3175.02 | 15642 | 86.75 | 10918 | 51.59 |
| **Carabobo** | 20 | 0.06 | 44 | 0.14 | 12 | 0.04 | 28669 | 100.36 | 10552 | 31.60 |
| **Cojedes** | 3 | 0.06 | 3 | 0.06 | 0 | 0.00 | 4015 | 108.50 | 1806 | 44.02 |
| **Delta Amacuro** | 0 | 0.00 | 0 | 0.00 | 28729 | 1267.16 | 1433 | 88.68 | 1605 | 85.12 |
| **Distrito Metrop** | 70 | 0.22 | 151 | 0.49 | 5 | 0.02 | 35736 | 135.71 | 9024 | 29.65 |
| **Falcón** | 1 | 0.01 | 10 | 0.08 | 0 | 0.00 | 10864 | 95.32 | 3767 | 28.77 |
| **Guárico** | 2 | 0.02 | 7 | 0.07 | 217 | 1.89 | 10954 | 117.05 | 4591 | 41.53 |
| **Lara** | 25 | 0.10 | 52 | 0.20 | 1 | 0.00 | 22274 | 97.51 | 8721 | 33.19 |
| **Mérida** | 9 | 0.08 | 10 | 0.09 | 129 | 1.13 | 11617 | 109.48 | 4116 | 33.54 |
| **Miranda** | 72 | 0.17 | 127 | 0.30 | 216 | 0.47 | 41862 | 121.33 | 8649 | 21.73 |
| **Monagas** | 3 | 0.02 | 9 | 0.08 | 10101 | 72.16 | 9559 | 88.15 | 5874 | 46.73 |
| **Nva. Esparta** | 3 | 0.05 | 9 | 0.13 | 25 | 0.41 | 5906 | 103.11 | 2465 | 37.07 |
| **Portuguesa** | 1 | 0.01 | 7 | 0.06 | 669 | 5.70 | 11085 | 101.96 | 5063 | 40.48 |
| **Sucre** | 18 | 0.13 | 26 | 0.19 | 74362 | 543.85 | 11245 | 96.88 | 4887 | 35.80 |
| **Táchira** | 21 | 0.13 | 26 | 0.16 | 321 | 2.00 | 19107 | 130.51 | 6029 | 35.45 |
| **Trujillo** | 10 | 0.10 | 14 | 0.14 | 49 | 0.48 | 13120 | 146.66 | 4206 | 40.42 |
| **Vargas** | 11 | 0.23 | 15 | 0.31 | 152 | 2.79 | 6195 | 140.15 | 1358 | 26.40 |
| **Yaracuy** | 3 | 0.03 | 3 | 0.03 | 0 | 0.00 | 8655 | 114.97 | 3529 | 40.24 |
| **Zulia** | 34 | 0.06 | 70 | 0.13 | 6363 | 11.43 | 57350 | 127.24 | 24351 | 47.05 |
| **1- CDBE: Conflict death best estimate, 2- CDHE: conflict death high estimate, 3- MAL: malaria incidence, 4-malaria rate, 5-HDD: heart diseases deaths, 6- heart disease mortality rate 7- IFU1D: infant under 1 deaths, 8- IFU1M: infant under 1 mortality rate** | | | | | | | | | | |
